# Network-Free Inference of Knockout Effects in Yeast

**DOI:** 10.1371/journal.pcbi.1000635

**Published:** 2010-01-08

**Authors:** Tal Peleg, Nir Yosef, Eytan Ruppin, Roded Sharan

**Affiliations:** 1 Blavatnik School of Computer Science, Tel-Aviv University, Tel-Aviv, Israel; 2 School of Medicine, Tel-Aviv University, Tel-Aviv, Israel; King's College London, United Kingdom

## Abstract

Perturbation experiments, in which a certain gene is knocked out and the expression levels of other genes are observed, constitute a fundamental step in uncovering the intricate wiring diagrams in the living cell and elucidating the causal roles of genes in signaling and regulation. Here we present a novel framework for analyzing large cohorts of gene knockout experiments and their genome-wide effects on expression levels. We devise clustering-like algorithms that identify groups of genes that behave similarly with respect to the knockout data, and utilize them to predict knockout effects and to annotate physical interactions between proteins as inhibiting or activating. Differing from previous approaches, our prediction approach does not depend on physical network information; the latter is used only for the annotation task. Consequently, it is both more efficient and of wider applicability than previous methods. We evaluate our approach using a large scale collection of gene knockout experiments in yeast, comparing it to the state-of-the-art SPINE algorithm. In cross validation tests, our algorithm exhibits superior prediction accuracy, while at the same time increasing the coverage by over 25-fold. Significant coverage gains are obtained also in the annotation of the physical network.

## Author Summary

Observing a complex biological system in steady state is often insufficient for a thorough understanding of its working. For such inference, perturbation experiments are necessary and are traditionally employed. In this work we focus on perturbations in which a gene is knocked out and as a result multiple genes change their expression levels. We aim to use a given set of perturbation experiments to predict the results of new experiments. Using a large cohort of gene knockout experiments in yeast, we show that the emerging map of causal relations has a very simple structure that can be utilized for the prediction task. The resulting prediction scheme, and its extension to more complex functional maps, greatly improve on extant approaches, increasing the coverage of known relations by 25-fold, while maintaining the same level of prediction accuracy. Unique to our approach is its independence of physical network data, leading to its high efficiency and coverage as well as to its wide applicability to organisms whose interactions have not been mapped to date. We further extend our method to annotate the interactions of a physical network as activating or suppressing, obtaining significant coverage gains compared to current approaches.

## Introduction

High-throughput technologies are routinely used to map molecular interactions within the cell. These include chromatin immuno-precipitation experiments for measuring protein-DNA interactions (PDIs) [1], and yeast two-hybrid assays [2] and co-immunoprecipitation screens [3] for measuring protein-protein interactions (PPIs). The resulting maps provide a scaffold from which one can extract regulatory-signaling mechanisms that underlie cellular processes and responses.

Physical interactions however may not be sufficient to deduce causal roles played by genes in regulation and signaling. For such deduction, perturbation studies are necessary and are traditionally employed [4]. Here, we focus on perturbation studies in which a gene is knocked out and as a result multiple genes change their expression levels. These measurements can be used to derive a functional map of genes, providing a complementary view to the physical one. While in the physical map an edge between two proteins (PPI) or between a protein and a gene's promoter sequence (PDI) indicates a direct association, in the functional map an edge connects two genes if knocking out one of them affects the expression level of the other.

The problem of explaining knockout experiments using a physical network was first introduced by [5]. The authors looked at a specific setting of the problem where the objective is to annotate each physical edge with the *direction* in which information flows through that interaction, and a *sign*, representing the regulatory effect of the interaction (activation or suppression). A followup work by Ourfali et al. [6] introduced the SPINE algorithm, aimed at annotating the physical network while maximizing the expected number of knockout effects that can be explained by the physical model. In both cases, the annotated physical network was used for predicting new knockout effects (up- or down-regulation).

Another line of work, related to the analysis of single knockout experiments, is the analysis of genetic interactions. Qi *et al.* [7] used a functional network of genetic interactions for inferring physical and genetic associations in yeast. They identified relations of complex/pathway co-membership with paths of even length in the functional network, whereas novel genetic relations were identified with odd-length paths. Segre *et al.* [8] studied a partition of the yeast metabolic system into groups based on patterns of aggravating and alleviating effects in response to double gene perturbations. The groups were constructed hierarchically so as to interact with each other monochromatically, i.e., with purely aggravating or purely alleviating effects across groups, enabling the authors to predict new genetic interactions.

Here we present a novel approach for analyzing a functional network to infer knockout effects. In contrast to previous work, our method does not depend on knowledge of a physical network, but in fact decouples the task of predicting knockout effects from the task of annotating the edges of the physical network. The method is based on partitioning the genes into functional groups whose members are indistinguishable with respect to the rest of the (functional) network.

We start by considering a partition of the genes into two “chromatic” groups with links of up-regulation between the groups and links of down-regulation within each group. To motivate this model, we show that if the latent physical network that underlies the functional data has no cycles with an aggregate negative sign (i.e., the product of the signs along the cycle's edges is negative), then such a partition is indeed possible. We devise several tests for the two-group assumption and find that it is sufficient to explain a large fraction of the analyzed data. Nevertheless, we find that negative feedback mechanisms within signaling pathways lead to deviations of the experimental data from this model. To tackle such deviations, we extend our algorithm to more than two groups, based on ideas from the work of [8] (described above).

We validate our methods using a collection of over two hundred knockout experiments in yeast [9]. We conduct cross validation experiments by hiding a subset of the resulting knockout pairs (of a deleted gene and an affected gene), and using the remaining pairs to predict the effects of the hidden pairs (up- or down-regulation). We attain high accuracy (88%) and coverage (73.8%) levels in the prediction task (when applying the extended algorithm). Moreover, the high efficiency of our algorithms allows us to analyze the entire data set in seconds. These results provide a substantial improvement over the state of the art SPINE algorithm [6], and over a previous benchmark by Yeang *et al.* [5]. In contrast to our approach, these methods are not “network-free”; instead they depend on a brute-force enumeration of all possible physical pathways between every knockout pair. Often times, such an enumeration is not feasible, which limits the applicability of these methods to gene pairs that are at most 3 edges apart in the physical network. In yeast, this limits the algorithms to a miniscule fraction of 4% of the knockout pairs available. Consequently, SPINE attains a coverage level of 2.6%, a 25-fold reduction in comparison to our method; at the same time, it also yields a lower accuracy (72%).

Finally, we tackle the task of annotating the physical edges with signs of activation or suppression. We provide an efficient algorithm for annotating a given physical network so as to explain a maximal number of functional relations. We validate the algorithm by using manual annotation of the filamentous growth pathway [10], and the high osmolarity glycerol (HOG) pathway [11]. Altogether, we obtain accuracy levels that are comparable to those of SPINE [6] while significantly improving on its coverage.

## Results/Discussion

We follow the seminal work of Yeang et al. [5] who aimed at explaining the results of knockout experiments using a physical (PPI and PDI) network. In each experiment a selected gene was knocked out, and the genome-wide expression response was measured. The basic paradigm of their work was that any knockout effect, i.e., the increase/decrease in expression of a certain gene following the knockout of another gene, can be explained via a physical pathway that connects the knocked out gene to the affected gene. Moreover, the aggregate influence of the interactions along the pathway should be equal to the complement of the observed effect. Consequently, they aimed at annotating the physical network with activation/suppression attributes so as to explain a maximal number of the observed effects. They used this annotation to predict new knockout effects.

Given a set of knockout experiments, we start by representing them as a *functional network* whose nodes are genes and edges connect gene pairs if knocking out one of them significantly changes the expression level of the other. The sign of an edge in the functional network complements that of the knockout effect (as it represents the wild type effect): “+” when the knockout results in down-regulation, and “−” otherwise. In the following we suggest a novel approach that utilizes the structure of this network in order to predict knockout effects. We evaluate our approach and compare it to the previous work of [5],[6] using a data set of 24,457 high confidence knockout pairs obtained from genome-wide expression measurements in yeast under 210 single-gene knockouts [5],[9].

### The sign-linear model

We say that a functional network is *sign-linear* if there exists a Boolean assignment 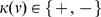 for every gene 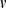 such that the sign of each edge 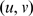 in the network is 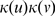 (a condition which can be cast in the form of a linear equation, hence the name of the model; see Methods). In this case we also say that 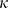 *explains* the input functional relations. Assuming that a given functional network is sign-linear essentially means that we can retain all the information from the knockout experiments by partitioning the genes into two groups. Gene pairs linked by a down-regulation edge in the functional network will be on the same group and pairs linked by an up-regulation edge will be on different groups.

To motivate this assumption, it is imperative to consider its implication on the physical network that underlies the observed knockout effects. We say that a physical network is *sign-consistent* if it does not contain an undirected cycle (i.e., any loop in the network when disregarding edge directions) with a negative aggregate sign (Methods). Notably, the sign-consistency assumption is reminiscent of the acyclicity assumption that is the basis for Bayesian modeling of biological networks [12],[13]. As we show in Text S1, a sign-consistent physical network implies a sign-linear functional network, and for every functional network, one can construct a sign-consistent physical network that explains it.

**Figure 1:**
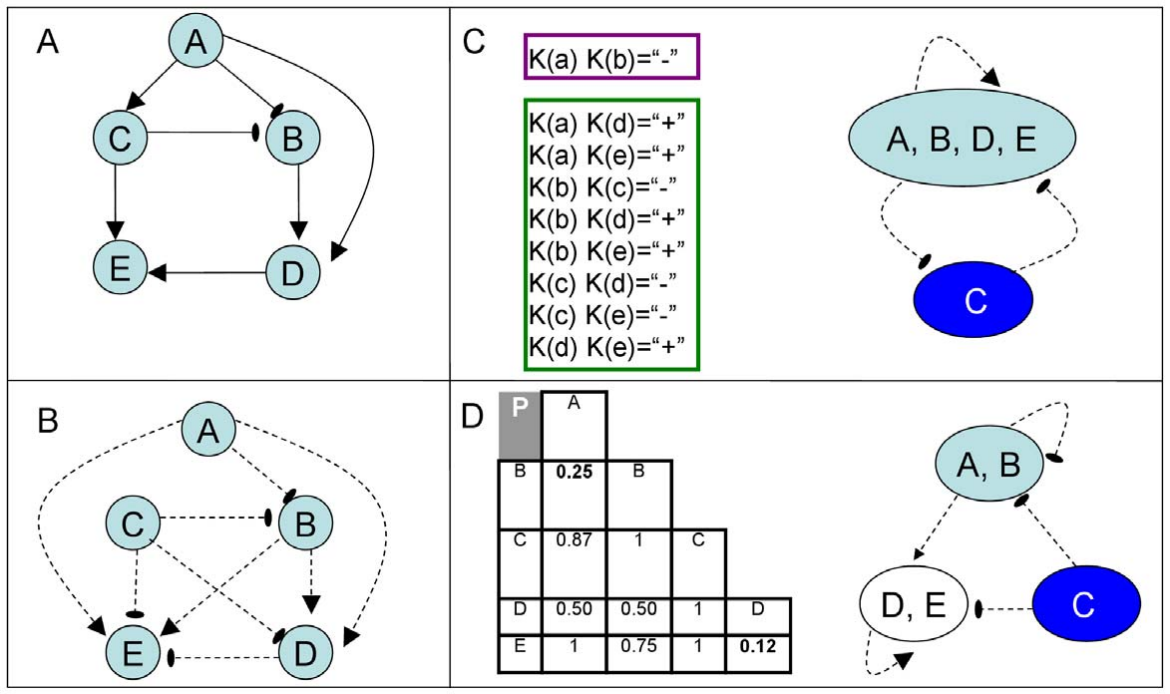
Algorithmic overview. (A) A physical network model with nodes representing proteins and edges representing protein-DNA interactions. The sign of an interaction is denoted by its arrow type: regular (activating) or cut (suppressing). Note that the network is not sign-consistent since for example, 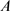 is linked to 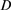 by two paths with different aggregate signs. (B) A functional network generated by the physical network (every knockout effect is explained by at least one path in the physical network, see Methods) with edges representing knockout effects and nodes representing the respective genes. The sign of a functional edge is denoted by its arrow type: regular (down-regulation) or cut (up-regulation). (C) The sign-linear algorithm. The functional network is translated into a set of Boolean equations. One optimal solution for the equations is setting 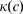 to 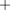 and the rest to 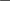, satisfying all equations (green frame, bottom) but one (purple frame, top). The ensuing partition into two groups is depicted with edges corresponding to functional relations between groups. This partition can be used for predicting new knockout effects. (D) The sign-clustering algorithm. For each pair of nodes the presented 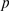-value reflects their similarity in the functional network. A partition into clusters using a cutoff of 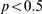 is depicted with edges defined as in panel C. This partition refines the one obtained by the sign-linear algorithm (3 groups instead of 2), correctly modeling all the knockout effects.

If a network is sign-linear then one can efficiently compute a Boolean assignment that explains the input functional relations, and the task of predicting a knockout effect translates to computing the product of the signs of the participating nodes. In the general case, such a perfect Boolean assignment might not exist. Instead, we aim to find an assignment that will satisfy as many of the observed functional relations as possible (see Methods and Figure 1). To tackle this computationally hard problem, we use an efficient randomized heuristic that is guaranteed to converge to a local maximum. Given a locally-optimal Boolean assignment 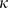, the sign of the effect of gene 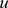 on gene 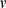 is predicted to be 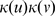. We run the randomized procedure multiple times, potentially obtaining different assignments 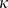, and compute a consensus assignment (Methods). It should be noted that the algorithm is restricted to genes that are implicated in at least one experiment (either as a knocked out gene or as an affected gene; see Methods).

We tested the validity of the sign-linearity assumption using the yeast knockout data. Applying a single iteration of the sign-linear algorithm to the entire data set, we obtained a Boolean assignment that satisfies over 83% of the knockout pairs (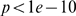, Text S1). This result indicates that the respective functional network is highly structured and can be readily utilized for predicting knockout effects under the sign-linear model.

### The yeast mating network benchmark

We use the yeast mating network, studied in [5],[6], as a first test case. The mating network contains 46 genes involved in pheromone response and 58 physical interactions (25 PPIs and 33 PDIs). The 46 genes span 149 (of 24,457) functional relations. Due to scalability problems, the application of both previous methods was limited to 103 of the functional interactions, considering only pairs of genes that are at most 5 edges apart in the physical network.

Two variants of SPINE [6] were employed for predicting the results of knockouts in the mating network, one that assigns signs to edges, and one that assigns signs to nodes (forcing all the edges that emanate from a node to carry its sign). We compare the performance of the sign-linear algorithm on the restricted set of 103 knockout pairs to the results of [5] and both variants of [6]. All algorithms were applied in a leave-one-out cross validation setting, each time hiding a single knockout pair and using the remaining ones to predict its outcome. The ensuing performance is evaluated using two quality measures: (i) *Accuracy*: the percentage of correct predictions out of all predictions made; and (ii) *coverage*: the percentage of knockout pairs that were predicted correctly out of the entire set of knockout pairs.

Table 1 summarizes the performance of the different approaches. While the best performance is achieved by [5] and the edge variant of [6], the accuracy and coverage of the sign-linear algorithm are only slightly lower. Importantly, our model employs a substantially simpler model with the number of variables being equal to the number of nodes, rather than to the number of edges (as in the other two models), making it less prone to over-fitting. Comparing the sign-linear model to the node variant of SPINE, which has an equivalent number of variables (one binary variable per gene), the sign-linear algorithm is found superior in both accuracy and coverage.

**Table 1:** Performance comparison in predicting knockout effects.

Method	Global Acc.	Global Coverage	Mating Acc.	Mating Coverage
Sign-linear	80.2%	76.4%	93.3%	92.2%
Sign-clustering	88.3%	73.8%	96%	94%
SPINE node variant	72.5%	2.6%	89.3%	89.3%
SPINE edge variant	NA	NA	99%	98%
Yeang *et al.* [5]	NA	NA	97.1%	97.1%

Shown are coverage and accuracy levels in predicting knockout effects using the entire knockout data (left) or focusing on the mating network (right). The results for the sign-linear and sign-clustering algorithms are presented for the most permissive decision cutoff (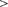
50%).

We further tested our method using varying sizes of the training set (leaving out 10%, 20% and 50% of the knockout pairs). The accuracy level remained stable at 90% even when leaving out 50% of the pairs. The coverage level was at 90% when leaving out 10% or 20% of the pairs, but dropped to 38% when leaving out 50% of the pairs.

### Genome-wide application

The simplicity of the model and the independence of physical data allows the sign-linear algorithm to be applied on large data sets on which the methods of [5] and [6] could not be applied. Considering the complete data set of 210 knockout experiments, the applications of [5] and [6] were confined to less than 4% (974) of the knockout pairs, for reasons of scalability. The limited set contained only pairs of genes that are at most 3 edges apart in the physical network. For the same reason, a cross-validation scheme similar to the one used for the mating subnetwork could not be applied with those algorithms, even with the limited data set. In contrast, the sign-linear algorithm could be tested in cross validation (each time leaving out 200 knockout pairs), and generated predictions for over 95% (23,312) of the pairs.

We compare the results of the sign-linear algorithm to results from [6], who applied the node variant of SPINE on the reduced data set without using cross validation (Text S1). The results in Table 1 show that the sign-linear algorithm outperforms SPINE both in accuracy (80.2% vs. 72.5%) and, more strikingly, in coverage (76.4% vs. 2.6%).

**Figure 2:**
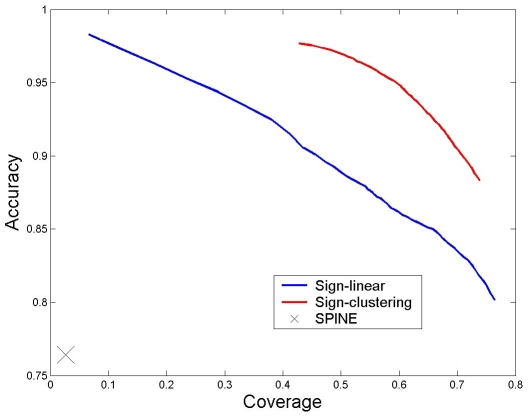
Accuracy versus coverage in the prediction of knockout effects on the genome-wide knockout data set. Results for the sign-linear and sign-clustering algorithms are displayed for different decision cutoffs. The results were obtained using cross validation, each time leaving out 200 knockout pairs. Results for SPINE are presented for its node variant as provided by [6], without using cross validation.

Thus far, we predicted a functional edge to be (for instance) *up-regulation* if the majority (more than 50%) of the obtained assignments implied so. Further probing the results of the sign-linear algorithm, we calculated the levels of accuracy and coverage obtained for more stringent decision cutoffs (*i.e.*, predict an effect only if a certain percentage (larger than 50%) of the assignments agree). Figure 2 plots the resulting accuracy-coverage curve. Evidently, the curve decreases monotonically, where for a coverage level of 10% we achieve over 98% accuracy. We also investigated the stability of the predictions across the different runs, observing that over half of the knockout pairs are predicted consistently by at least 90% of the runs (Figure S2).

Finally, we tested the robustness of the sign-linear algorithm to noise in the input data. Following [6], we flipped 5%, 10% and 15% of the input signs and applied the sign-linear algorithm to the perturbed data. The algorithm was highly consistent in its predictions, maintaining consistency levels of 93.3%, 90.1% and 86% under the different noise levels.

### Going beyond sign linearity

**Figure 3:**
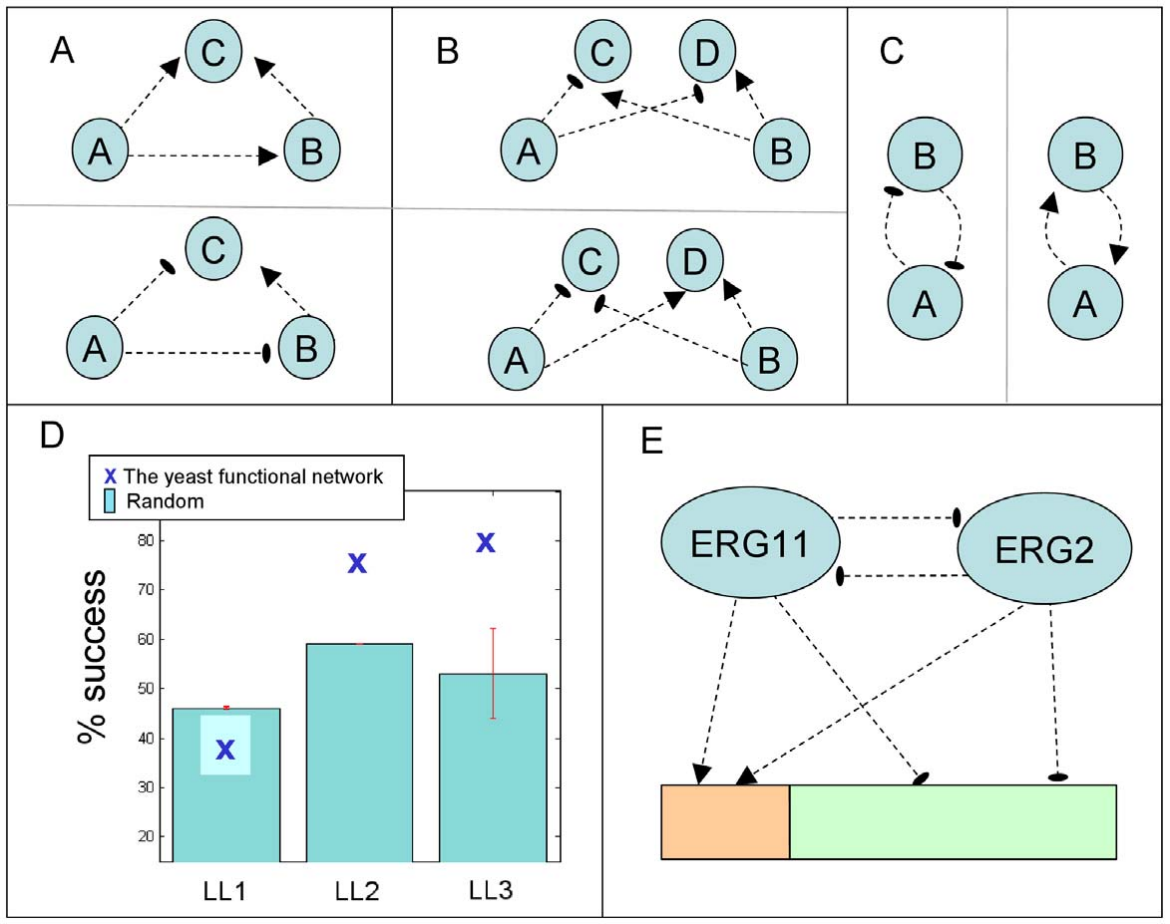
Evaluating local linearity properties of the functional network. Edges represent functional relations with down-regulation relations depicted as regular arrows and up-regulations as cut-arrows. (A) LL-1: if knocking out genes 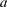 or 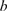 has a similar (opposite) effect on a shared target 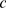, then if 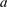 affects 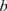 the relation should be down-regulation (up-regulation). (B) LL-2: for two knocked out genes 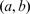 with at least two common targets 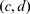, the respective influences should be either equivalent (bottom) or the exact opposite (top). (C) LL-3: If two genes 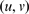 affect each other then the effects should have equal signs. (D) The prevalence of the three properties in the original data and in randomized networks. (E) An example for the violation of LL-1 in the biosynthesis of steroids pathway. Two pathway members, ERG11 and ERG2, that increase each other upon knockout, have the exact same effect on all their common targets, down-regulating 110 genes (orange rectangle) and up-regulating 308 genes (green rectangle).

While the sign-linear algorithm gave promising results, its underlying assumption is quite restrictive and about 20% of the data do not follow it. To characterize the deviations from the linearity assumption in a finer manner, we devised several local linearity tests for the following properties: (i) Local linearity 1 (LL-1) occurs when the effects of two knocked out genes on a common target is consistent with their effect on each other (Figure 3a). (ii) LL-2 entails that two different knocked-out genes should have the exact same influence on all of their common targets or the exact opposite influence (Figure 3b). (iii) LL-3 requires symmetry, *i.e.*, if two genes affect each other then the effects have to be equal (Figure 3c). Notably, the three tests represent all the ways in which a contradiction to the sign-linearity property can be reached with at most two knockout genes and two affected genes (Text S1).

We evaluated the prevalence of these three properties in the yeast knockout data set and compared the results to those obtained on randomized data sets (Text S1). The results in Figure 3d show that the regularities represented by LL-2 and LL-3 are indeed more prevalent than the random expectation. On the other hand, it is apparent that LL-1 is significantly less prevalent than in random. A possible explanation for the deviation from LL-1 may be the prevalence of signaling pathways in our data. It is reasonable to hypothesize that knocking out different components of the same pathway will result in deprivation of similar substrates and consequently generate a similar cellular response. Furthermore, the cellular response might utilize negative feedback mechanisms for activating the malfunctioning pathway by increasing the expression levels of the respective genes (rather than reducing it, as expected by LL-1; see Figure 3e). To provide support for these hypotheses we examined knockout profiles of components in manually curated pathways from the KEGG database [14]. For each pair of knocked out genes that are members of the same pathway we checked how many of their common targets are affected in the same manner. We found that genes in the same pathway indeed tend to affect the same genes (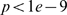), have similar effects on their common targets (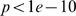), but increase each other's expression when knocked out (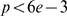). Similar results were obtained for genes that co-reside in the same MIPS [15] complex (data not shown).

One particular example is the biosynthesis of steroids pathway (*KEGG:sce00100*). Out of the 23 genes in the pathway we consider a subset of nine genes that were knocked out in [9]. Overall there are 26 knockout pairs involving these genes where all of the respective effects are up-regulation. The performance of the sign-linear algorithm in predicting these effects is understandably low, due to the violation of the LL-1 property, with 20 of the 26 effects wrongly assigned as down-regulation (notably, due its limited applicability, SPINE could not generate predictions for any of the knockout pairs within this set). The algorithm we present next uses a more flexible (albeit more complex) model designed to account for the under representation of the LL-1 property and to correctly model the relations exhibited within signaling pathways.

### The sign-clustering algorithm

A natural extension of the sign-linear model is to partition the genes into multiple (greater than two) groups, and use this as a baseline for predicting knockout effects. Taking an approach similar to [8], we assign the genes into groups by clustering together genes that are functionally similar. For a given pair of genes, our measure of functional similarity reflects both the similarity in their response to knockouts as well as the similarity of their effects on other genes when knocked out themselves (Methods).

The *sign-clustering* algorithm (Methods, Figure 1D) constructs the groups using a (randomized) hierarchical clustering procedure. Denote by 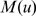 the group to which 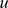 is assigned. To predict the effect of (knocking out) gene 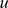 on gene 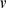, the effects of genes from 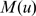 on genes from 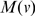 are considered. The prediction is made according to the majority of the considered effects (Methods); if no such effects were observed, the prediction is left undecided. Similar to the sign-linear algorithm, we run the clustering procedure multiple times, potentially obtaining different partitions, and compute a consensus prediction (Methods). Notably, the algorithm does not explicitly determine the number of groups. Instead, it uses a top-down procedure of iteratively partitioning the genes, until a certain stopping criterion is met. The partitioning is stopped when the concordance between the genes of the current candidate group is higher than the chance expectation (Methods). While the obtained groups do not necessarily correlate with densely connected regions of the physical network, almost half of them (49%) are functionally coherent with respect to the gene ontology (GO) annotation (see Text S1 for functional coherency computation). This is expected as these groups contain genes with similar functional relations according to the knockout data.

The sign clustering algorithm was applicable to over 83% (20,445) of the knockout pairs. The sizes of the resulting clusters varied from 1 to 35 with an average size of 4.5 (Figure S1). The algorithm attained an accuracy level of 88.3% and a coverage level of 73.8% (Table 1). Considering more stringent decision cutoffs as before, the resulting accuracy-coverage curve (Figure 2) points to a clear advantage in comparison to the sign-linear algorithm. The stability of the predictions over the different runs was similar to that of the sign-linear algorithm (Figure S2). The robustness to noise was slightly lower (consistencies of 88.2%, 86.7% and 84.3% when flipping 5%, 10% and 15% of the input signs, respectively). Zooming in on the biosynthesis of steroids pathway, we see that the sign-clustering algorithm correctly captures the respective functional relations. It predicts correctly 24 out of 26 effects where in 17 of the cases the correct prediction was made unanimously by all the computed partitions.

### Annotating the physical network

The partition into functional groups introduced above can also facilitate the annotation of edges in a physical network with signs of activation or suppression. Given a physical network, hypothesized to provide the underlying “wiring” for the knockout effects, the problem of assigning signs (“+” for activation and “−” for suppression) on its edges so as to explain a maximum number of knockout pairs is computationally hard (Text S1). We present a novel algorithm for this problem that determines the sign of a physical edge between two proteins according to the functional relations between the groups of their respective genes, associating “negative” functional relations (up-regulation) with “negative” physical interactions (suppression) and vice versa (Text S1). In the following we concentrate on partitions into two groups 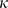, where the algorithm predicts a physical edge from node 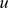 to 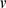 to be 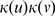. As before, we use multiple Boolean assignments and compute a consensus prediction.

**Figure 4:**
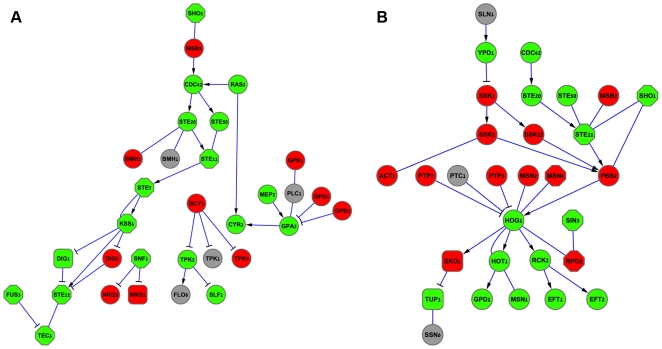
Annotating physical interactions with signs of activation or suppression. The filamentous growth pathway in yeast is displayed in frame A; The high osmolarity glycerol (HOG) pathway is displayed in frame B. Literature curated interaction signs are denoted by the arrow type: regular (activating), cut (suppressing), or none (unassigned). Node colors correspond to a specific partition of the respective genes into two groups made by the sign-annotation algorithm. Gray nodes represent proteins that could not be assigned to a group due to a lack of data. Physical edges connecting proteins of different groups are predicted as suppressing, and edges connecting proteins of the same group are predicted as activating. SPINE, in contrast, assigns signs to proteins, meaning that all the out-going edges of a protein are assigned the same sign. Proteins that were predicted by SPINE to be activators are displayed as hexagons. Proteins that were predicted by SPINE as suppressors are displayed as squares.

We constructed a network of physical interactions in yeast, containing 5,850 nodes, and 45,512 interactions (39,946 PPIs and 5,566 PDIs), using information from public data bases [16],[17] and from large scale assays [1],[3],[18],[19]. We annotated the network using the knockout data. Altogether, the algorithm annotated 74% of the edges as activating or suppressing. We validate these predictions using manual annotations of the filamentous growth pathway [10] and the high osmolarity glycerol (HOG) pathway [11]. Figure 4 depicts the annotation of the two pathways by our method and by SPINE. Comparing to the literature benchmark, our algorithm obtained an accuracy of 75% and coverage of 69% in predicting signs in the filamentous growth pathway; and an accuracy of 72% and coverage of 65% with respect to the HOG pathway. These results compare favorably with those of SPINE [6], which attained accuracy levels of 44% and 100% and coverage levels of 15% and 10% for the filamentous growth pathway and the HOG pathway, respectively.

One interesting finding of our algorithm concerns the annotation of the interactions between the suppressor of sensor kinase 2 (Ssk2) and Actin 1 (Act1) in the HOG pathway. While the manual annotation of this edge [11] is undecided, the algorithm predicted it to be stimulatory (activating). This finding is in line with evidence that Ssk2 is required for the actin reassembly and for the recovery from osmotic stress. While the mechanism behind this dependency is not clear, it was previously suggested that actin is a potential substrate of the Ssk2 kinase [20].

### Conclusions

We devised two clustering methodologies for predicting knockout effects based solely on a given network of functional interactions. The first algorithm employs a restrictive assumption on the structure of the functional network; nevertheless, its underlying model is sufficient for describing the majority of the knockout effects in the large scale yeast data set that we analyzed. In cross validation tests it was shown to provide very efficient means for predicting held-out knockout effects, dramatically improving upon the state-of-the-art benchmark. The second, refined algorithm extends the two-group logic that is at the heart of the first algorithm, aiming to partition the genes into several groups that behave similarly with respect to the knockout data. We show that this refined model allows capturing functional relations within signaling pathways, which could not be explained by the previous model, leading to superior accuracy.

Notably, since the input data contains only single-gene perturbations, both algorithms cannot decipher combinatorial regulation functions involving multiple inputs (as in [4]). Instead, the algorithms treat the functional relations independently and try to find the best way to consolidate them (*i.e.*, maximizing the number of relations that can be explained by the model).

Being “network-free” (*i.e.*, independent of physical interaction data) is a unique feature of our algorithms, which allows their application to organisms on which no comprehensive interaction data is available. To complement the analysis when a physical network is available, we show how to use the information embedded in a functional network to annotate the physical edges with signs of activation or suppression. In comparison with a previous method, our algorithm is again shown to provide a substantial improvement in terms of coverage while attaining comparable levels of accuracy.

In a recent paper, Ma'ayan *et al.* [21] studied the prevalence of sign-consistent versus sign-inconsistent loop motifs in the yeast physical regulatory network. Their findings suggest that sign-consistent loops are more prevalent and that, overall, the network is close to being sign-consistent. Our work provides further support to this observation through the results of the local linearity tests and the overall good performance of the sign-linear model on the yeast data. It will be interesting to test how well do gene perturbation maps in higher organisms conform to the simplistic sign-linear model. As data from perturbation experiments in human gradually accumulates [22], this is an appealing direction for future research.

## Materials and Methods

We define a *functional network* as a directed graph whose nodes are genes and whose edges connect gene pairs 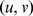 if knocking out 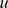 changes the expression level of 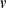. The *sign* of an edge, denoted 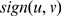, is opposite to the effect of the respective knockout (“+” if knocking out 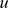 down-regulates 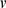 and “−” if 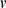 is up-regulated). We define the *aggregate* sign of a given subgraph as the product of the signs along its edges.

### Physical models of sign-linear functional networks

Let 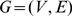 be a connected, directed network of physical interactions. We denote by 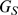 the network 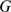 annotated with signs 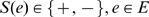 on its edges. The *undirected form* of 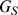 is an undirected graph of the same topology as 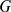 whose edges are annotated according to 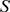. In case there are contradicting signs: 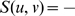, 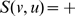, then the undirected form of 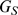 is not defined. We say that an annotated network 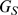 is *sign-consistent* if its undirected form is defined and does not contain cycles with a negative aggregate sign.

Let 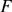 be a functional network defined on a subset of the nodes in the physical network 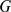. An edge 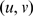 in 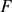 is *explained* by the annotated network 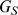 if and only if there exists a path in 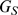 from 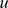 to 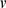 such that its aggregate sign is equal to the sign of the knockout relation 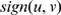. Similarly, we say that 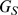 can *generate* the relation 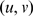. We say that 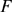 can be explained by 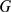 if there exists a Boolean assignment 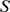 such that 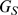 can explain all the knockout effects in 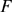. Similarly, we say that 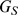 can generate 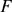 if it explains all the edges in 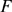.

The following two lemmas motivate our sign-linear algorithm; their proofs appear in Text S1.

#### Lemma 1

*A sign-consistent annotated physical network can only generate sign-linear functional networks.*

#### Lemma 2

*If* 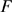 *is sign-linear then for every connected physical network* 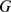 *defined on a super set of the nodes in* 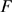 *, there exists an assignment* 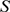 *such that* 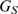 *is sign-consistent and explains* 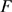.

### The sign-linear algorithm

The sign-linear algorithm is based on finding a Boolean assignment 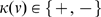 for every gene 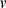 in the functional network that maximizes the number of knockout pairs 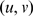 such that 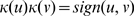. This maximization problem is also known as MAX-E2-LIN2, and can be reformulated in a set of linear equation in the space 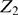. An approximation algorithm to MAX-E2-LIN2 was previously presented [23], however, for reasons of simplicity and scalability we chose to use a greedy approach. The latter starts from a random Boolean assignment and proceeds by choosing a gene at random and changing its assignment if it improves the result (*i.e.*, if it increases the number of explained pairs). The algorithm terminates when it reaches a local maximum, and no more modifications can be made. We predict the sign of a hidden knockout effect 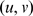 as 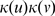. We repeat this randomized procedure 100 times and report the percentage of runs that predicted up- or down-regulation. Notably, the algorithm is only applicable to pairs of genes that lie in the same connected component of the (undirected) functional network.

### The sign-clustering algorithm

To obtain general partitions into more than two groups we use a hierarchical clustering procedure. For a given pair 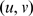, let 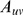 be the set of genes whose knockout affected both 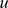 and 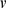, and let 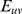 denote the set of genes that are affected by the knockout of 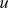 and by the knockout of 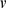 (this set is not empty only if the data set includes a knockout of 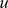 and a knockout of 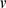). Let 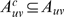 be the set of genes whose knockout affected 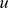 and 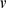 in a similar manner. Similarly, let 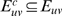 comprise of genes who responded similarly to the knockouts of 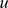 and 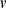. The pairwise similarity score that we use for the clustering procedure is calculated using a binomial cumulative distribution function 
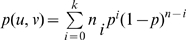
 where 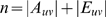 is the number of trials, and 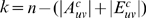 is the number of “failures” (namely, the number of times 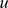 and 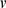 behaved differently). The resulting score is the probability of observing up to 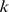 failures in 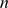 independent trials. The probability of a failure in any given trial is set to 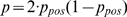, where 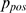 is the frequency of “+” relations in the functional network.

We use a standard complete-linkage hierarchical clustering procedure. We define the groups by finding inner nodes in the hierarchy whose score is lower than the a-priori probability for functional similarity (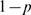) and the score of their ancestors in the hierarchy is larger than 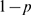. We predict the sign of a hidden knockout effect 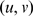 according to the groups 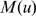 and 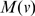 to which 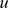 and 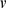 were mapped. If in the majority of the cases knocking out members of 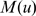 decreases members of 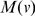, then 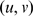 is predicted as down-regulation and vice versa. Due to its greedy nature, the order in which the genes are processed by the clustering procedure can affect the resulting clusters. Therefore, we repeat the procedure using 100 random orderings, and report for each pair the percentage of runs in which its relation was predicted to be up- or down-regulation.

## Supporting Information

Text S1 Supporting Information
(0.20 MB PDF)

Figure S1 Distribution of the sizes of clusters constructed by the sign-clustering algorithm.
(0.05 MB JPG)

Figure S2 The number of predictable knockout pairs as a function of the decision cutoff
(0.06 MB JPG)
