# Supplementary material for: Network-Free Inference of Knockout Effects in Yeast
Source: PLoS Comput Biol. 2010 Jan 8;6(1):e1000635. doi: 10.1371/journal.pcbi.1000635 (PMC2795781; doi:10.1371/journal.pcbi.1000635)
Supplement: Text S1 — Supporting Information (0.20 MB PDF) [file pcbi.1000635.s001.pdf]

Network-free Inference of Knockout Effects in Yeast - Text S1

Tal Peleg *et al.*

Physical models of sign-linear functional networks

Lemma 1 *A sign-consistent annotated physical network can only generate sign-linear functional networks*

Proof: Let G_S be a sign-consistent annotated physical network and let F be some functional network generated by G_S. We prove the claim by showing how to divide the proteins into two groups such that proteins from the same group can only have paths with positive sign between them, and proteins from different groups can only have paths with negative sign between them. Consider a random protein a in G_S. For each protein b consider some path between a and b in the undirected form of G_S. We assign on all the proteins that have positive paths from a (including a) as one group, and all the others as the second group. For proteins b, c in the same group, if they have negative path between them in G_S, then it follows that the undirected form of G_S necessarily contains a negative cycle (going from a to b, from b to c and from c to a) which contradicts the sign-consistency of G_S. Similarly, proteins b, c in different groups can not have positive path between them.Therfore,any generated functional network must be sign-linear . ■

Lemma 2 *If F is sign-linear then for every connected physical network G defined on a super set of the nodes in F there exists an assignment S such that G_S is sign-consistent and explains F*

Proof: Since F is sign-linear then there exists a Boolean assignment $\kappa(a) \in \{+, -\}$ such that for every edge (u, v) we get $sign(u, v) = \kappa(u)\kappa(v)$. Given a connected physical network G, defined on a super set of the nodes in F, we will construct an assignment S such that G_S is sign-consistent and explains F.

Consider a cut in G with all the nodes with a boolean value "+" on one side, all the nodes with the value "-" on the other size, and with the remaining nodes arbitrarily partitioned between the two cuts. For edges (u, v) crossing the cut we define $S(u, v) = " - "$ and for the remaining edges we set $S(u, v) = " + "$. Any undirected cycle will pass an even number of times in the cut, and therefore will have a positive sign. Every path between two proteins from the same group will have a positive sign, and a path between proteins from different groups will have a negative sign. It therefore follows that S can explain every knockout pair (u, v) in F and that S is sign-consistent. ■

Local-linearity tests

Let $\kappa(x) \in \{+,-\}$ be a Boolean assignment of a gene x, and let $\text{sign}(x,y) \in \{+,-\}$ be the effect of knocking out x on the expression of y (+ when the knockout results in down-regulation, and $-$ otherwise).

- LL-1: for every 3 genes a, b, c if the edges (a,b), (b,c) and (a,c) exists in the functional network, then LL-1 holds true if $sign(a,b) \cdot \text{sign}(b,c) = \text{sign}(a,c)$.
- LL-2: for every four-tuple of nodes a, b, c, d, if the edges (a,c),(a,d),(b,c) and (b,d) exists in the functional network, then LL-2 holds true if $\text{sign}(a,c) = \text{sign}(b,c)$ and $\text{sign}(a,d) = \text{sign}(b,d)$,or $\text{sign}(a,c) \neq \text{sign}(b,c)$ and $\text{sign}(a,d) \neq \text{sign}(b,d)$.
- LL-3: for every node pair a, b, if the edges (a,b), (b,a) exists in the functional network, then LL-3 holds true if $\text{sign}(a,b) = \text{sign}(b,a)$.

The results reported in Figure 3d are the percentage of feasible cases (where the respective functional relations exists) in which each LL property holds.

Lemma 3 *The three local-linearity tests represent all the ways in which sign-inconsistency can be reached with at most two knockout genes and at most two affected genes.*

Proof: Let a, b be the knocked-out genes and c, d the target genes. There are 6 possible functional edges/relations in the a, b, c, d system: $a \to b$, $b \to a$, $a \to c$, $a \to d$, $b \to c$, $b \to d$, where $x \to y$ means that knocking out gene x effected the expression of gene y. The sign of the functional edges $\text{sign}(x,y)$ is determined according to the direction of the effect, as defined above. Considering a Boolean assignment function κ, each functional relation can be represented by an equation $\kappa(x)\kappa(y) = \text{sign}(x,y)$.

If the system is sign-consistent then an assignment κ exists such that all the relevant equations are satisfied. The only way a contradiction to the existence of such an assignment can be obtained is through loops in the functional network that have an aggregate negative sign. The complete list of contradicting loops (up to replacing a with b and c with d) is:

- 2-edge negative loop: $\text{sign}(a,b) \cdot \text{sign}(b,a) = -1$
- 3-edge negative loop: $\text{sign}(a,c) \cdot \text{sign}(a,b) \cdot \text{sign}(b,c) = -1$
- 4-edge negative loop: $\text{sign}(a,c) \cdot \text{sign}(b,c) \cdot \text{sign}(a,d) \cdot \text{sign}(b,d) = -1$

From the definition above it can be seen that forcing a positive 2-edge loop is equivalent to LL-3; forcing a positive 3-edge loop is equivalent to LL-1, and forcing positivity on 4-edge loop is equivalent to LL-2. ■

Statistical analysis

We compute the prevalence of sign linearity in the functional networks (either the LL properties or globally, looking at the fraction of edges satisfied by the sign-linear model) by comparing to randomized instances. We examined two modes of randomization. First, we shuffled the signs on the edges of the functional network. Second, we shuffled the edges in the functional network, while maintaining the original incoming and outgoing degrees and the amount of ‘’+” and ‘’-” functional edges for each gene. The reported p-values were computed by comparing the true result to the distribution obtained from the random instances assuming normal distribution. We obtained similar results in both cases and thus only reported the former.

We evaluated the tendency of members in signaling pathways to co-effect shared targets by computing a Jaccard coefficient for each pair of genes in the pathway (defined as the size of intersection of their target sets divided by the size of their union) and comparing the results to the entire population of pairs (of knocked out genes) using a Wilcoxon ranksum test. The tendency to co-effect the shared targets similarly (activating or repressing) is computed in a similar manner, using the percentage of similarly affected targets (out of all shared targets) as the compared value. To evaluate the tendency of genes in a pathway to up-regulate each other upon knockout, let n be the number of knockout pairs from the same pathway and x be the number of pairs from this set where the effect is down-regulation. The reported p-value is computed using the cumulative binomial distribution function $\sum_{i=0}^{x} \binom{n}{i} p_{pos}^{i} (1 - p_{pos})^{n-i}$, where p_{pos} is the frequency of down-regulation interactions in the functional network.

We evaluate the functional enrichment of the groups obtained in the sign-clusterigng algorithm using the the gene ontology (GO) annotation system [1]. The enrichment of each GO term within a given group is computed by a hypergeometric p-value. The computation of p-values and their adjustment to multiple hypotheses is done using the GoTermFinder software [2]. We report the fraction of groups with at least 3 inner genes that were significantly coherent (corrected p-value lower than 0.05).

Application of SPINE

We use results from the application of the node-variant of SPINE, where the task in hand was to predict a fixed status of activation/ repression for each protein [3]. Following the construction presented in [3], a knockout pair was considered to be explained by a path in the physical network if all the proteins along the path are confidently annotated with a status of activation/ repression, and if the path obeys certain consistency rules defined in [3]. A given knockout pair is then considered to be predicted successfully if the expected number of explanatory paths that have the same aggregate sign as the true effect exceeded the expected number of paths with the opposite sign.

Annotating the physical map

Given a physical network G, and a functional network F, the sign-annotation algorithm starts by applying the sign-linear or the sign-clustering algorithms to find a partition of the proteins in F into groups. The algorithm decided the status (activating or suppressing) of each edge (u, v) in G, according to the groups $M(u)$ and $M(v)$ to which u and v were mapped. If the majority of knockout relations between proteins from the two sets $M(u)$ and $M(v)$ is down-regulation then (u, v) is predicted as activating and vice versa. This is equivalent to the prediction of the knockout effect of protein u on protein v. As before, we repeat this procedure 100 times with different runs of the grouping algorithm and decide using a majority vote across all runs.

The maximal sign assignment problem

Let $G = (V, E)$ represent a network of physical interactions. Let F be a functional network as defined on a subset of the nodes in G. The objective of *maximal sign assignment* is to find an assignment S such that the annotated physical network G_S explains a maximal number of edges in F.

Theorem 4 *The problem of* maximal sign assignment *is NP-complete and is hard to approximate to within a ratio of 11/12*

Proof: We reduce from MAX-E2-LIN2 [4]: let X be the variables and Eq be the equations. We construct a physical network $G = (V, E)$, such that $V = (X \cup \{o\})$, and $E = \{(o, v_i)|x_i \in V\}$. We define a functional network F as follows: For each equation $v_i v_j = b$, we add an edge (v_i, v_j) to F with sign b. After finding the signs in G,we will give every variable x_i a value according to the sign on the edge (xi, o) (0 for ‘’+” and 1 for ‘’-”). It is easy to see that each solution to the maximal sign assignment problem implies a solution with the same value to MAX-E2-LIN2, and vice versa. Therefore the reduction is approximation preserving, implying hardness of approximation to within 11/12 [4]. ■

Supporting Figures

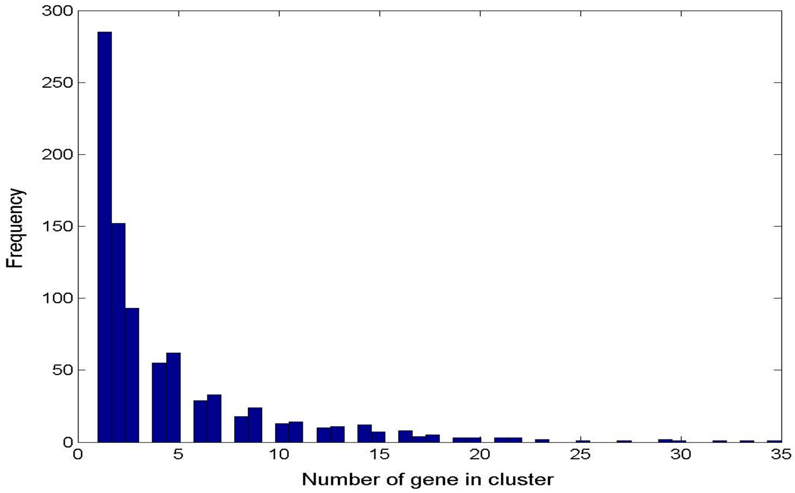

Figure 1: Distribution of the sizes of clusters constructed by the sign-clustering algorithm. The results are shown for a specific partition generated for the entire yeast knockout data set.

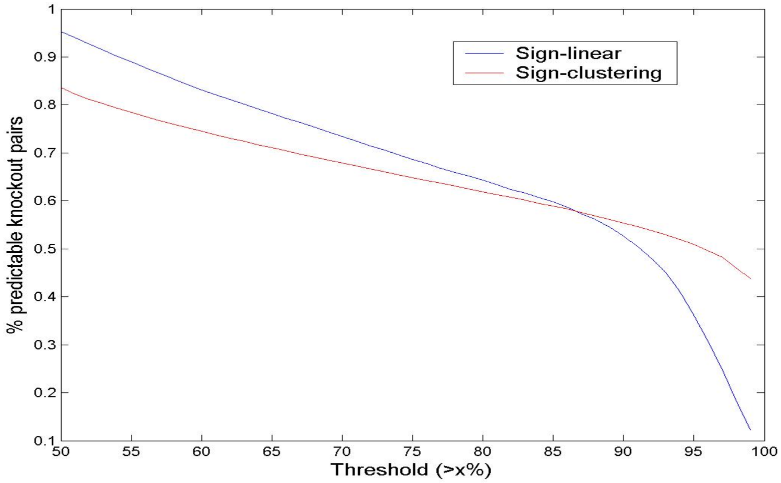

Figure 2: The number of predictable knockout pairs as a function of the decision cutoff (percentage of consistent runs required to make a prediction). Notably, for both methods, over half of the knockout pairs are predicted consistently by at least 90% of the runs.

References

[1] Harris M, Clark J, Ireland A, Lomax J, Ashburner M, et al. (2004) The Gene Ontology (GO) database and informatics resource. Nucleic Acids Res 1: D258–D261.

[2] Boyle E, Weng S, Gollub J, Jin H, Botstein D, et al. Gotermfinder. http://go.princeton.edu/cgi-bin/GOTermFinder.

[3] Ourfali O, Shlomi T, Ideker T, Ruppin E, Sharan R (2007) SPINE: a framework for signaling-regulatory pathway inference from causeeffect experiments. Bioinformatics 23: 359–366.

[4] Håstan J (2001) Some optimal inapproximability results. Journal of the ACM 48: 798-859.
