# Supplementary figures and images for: Network-Free Inference of Knockout Effects in Yeast
Source: PLoS Comput Biol. 2010 Jan 8;6(1):e1000635. doi: 10.1371/journal.pcbi.1000635 (PMC2795781; doi:10.1371/journal.pcbi.1000635)

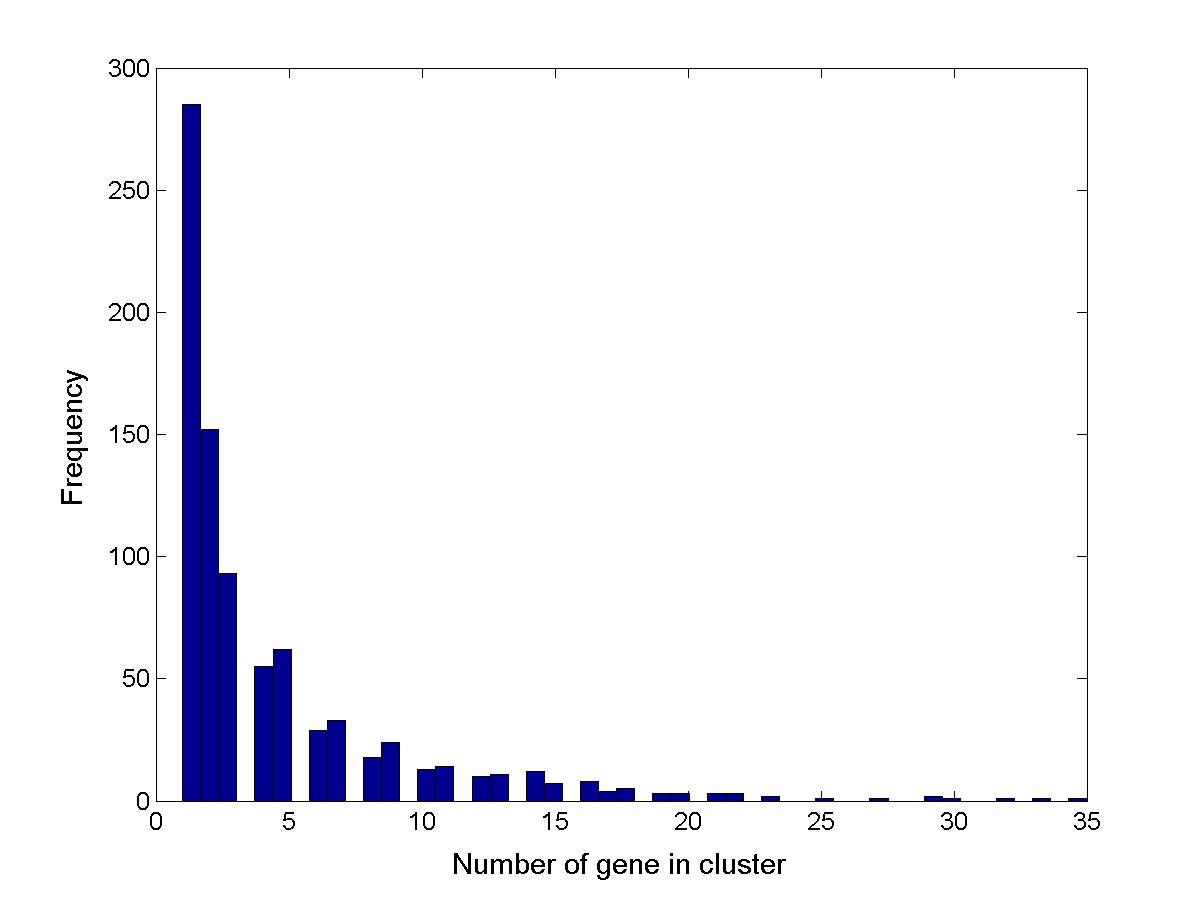

Supplement: Figure S1 — Distribution of the sizes of clusters constructed by the sign-clustering algorithm. (0.05 MB JPG) [file pcbi.1000635.s002.jpg]

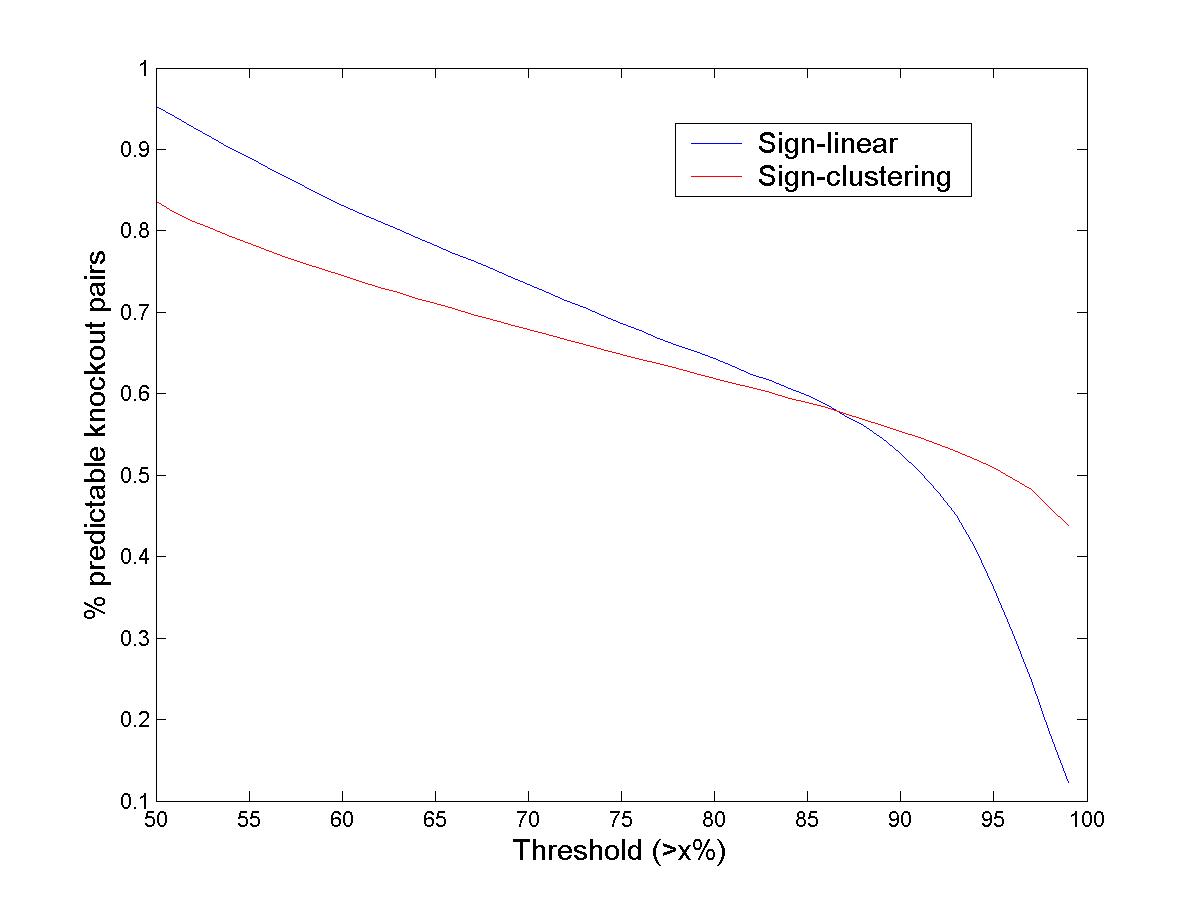

Supplement: Figure S2 — The number of predictable knockout pairs as a function of the decision cutoff (0.06 MB JPG) [file pcbi.1000635.s003.jpg]
